# Supplementary material for: Antibiotic Prescription and In-Hospital Mortality in COVID-19: A Prospective Multicentre Cohort Study
Source: J Pers Med. 2022 May 26;12(6):877. doi: 10.3390/jpm12060877 (PMC9224767; doi:10.3390/jpm12060877)
Supplement: Supplementary file 1 [file jpm-12-00877-s001.zip › jpm-1689340-supplementary.pdf]

Journal of Personalized Medicine

**Table S1:** The distribution of the variables in the survivors vs. non-survivors groups.

| Variable | Survivors, N = 505 | Non-Survivors, N = 48 | AUROC (CI 95%)* or RR (CI 95%)** | *p* Value | Missing |
|---|---|---|---|---|---|
| Symptoms of bacterial infection at admission, N (%) | 161 (31.9) | 9 (18.8) | 0.5 (0.2-1.04) | 0.071 | |
| Number of ATBs / patient, N (min, max) | 1 (1-7) | 2 (1-5) | 0.655 (0.570-0.740) | **<0.001** | |
| Length of ATB, median (min, max) | 10 (2-62) | 7 (2-26) | | 0.132 | 9 |
| ATBs-days from admission, median (min, max) | 0 (0-24) | 1 (0-20) | | 0.056 | 4 |
| ATBs-days from symptoms onset, median (min, max) | 8 (0-78) | 11 (0-40) | | **0.023** | 8 |
| Antifungal drugs, N (%) | 22 (4.4) | 4 (8.3) | | 0.270 | |
| Antiviral | 11 (22.9) | 179 (35.4) | | 0.111 | |
| Remdesivir, N (%) | 115 (22.8) | 9 (18.8) | 0.8 (0.4-1.6) | 0.591 | |
| Favipiravir, N (%) | 82 (16.2) | 4 (8.3) | | 0.209 | |
| Umifenovir, N (%) | 3 (0.6) | 0 | | 1 | |
| Ferritin at admission, median (min, max) | 455 (6-4787) | 761.5 (59-5421) | 0.640 (0.554-0.726) | **0.003** | 74 |
| Ferritin at ATB prescription, median (min, max) | 497 (6-4787) | 894 (59-5887) | 0.627 (0.508-0.746) | **0.042** | 240 |
| Procalcitonin at admission, median (min, max) | 0.09 (0.02-45.5) | 0.11 (0.03-24.8) | 0.575 (0.461-0.690) | 0.171 | 201 |
| Procalcitonin at ATB prescription, median (min, max) | 0.1 (0.02-23) | 0.16 (0.02-24.8) | 0.636 (0.523-0.748) | **0.019** | 245 |
| Eosinophils at admission, median (min, max) | 10 (0-1000) | 10 (0-300) | | 0.829 | 8 |
| Eosinophils at ATB prescription, median (min, max) | 10 (0-1270) | 30 (0-340) | | 0.526 | 62 |
| Platelet at admission, median (min, max) | 222 (27-817) | 197 (22-616) | 0.571 (0.485-0.657) | 0.104 | 8 |
| Platelet at ATB prescription, median (min, max) | 239.5 (27-817) | 205 (22-616) | 0.610 (0.510-0.709) | **0.036** | 280 |
| AST at admission, median (min, max) | 34 (9-548) | 50 (20-130) | 0.624 (0.540-0.708) | **0.005** | 20 |
| ALT at admission, median (min, max) | 30.6 (5-503) | 46 (9-179) | 0.606 (0.524-0.688) | **0.017** | 13 |
| Creatinine at admission, median (min, max) | 0.88 (0.4-5) | 0.98 (0.4-2.2) | 0.562 (0.467-0.657) | 0.156 | 8 |
| Sodium levels at admission, median (min, max) | 138 (109-157) | 137 (126-145) | | 0.423 | 10 |
| LDH at admission, median (min, max) | 303 (101-1319) | 365 (132-1113) | 0.611 (0.525-0.696) | **0.015** | 48 |
| Hospitalisation length (days), median (min, max) | 13 (2, 50) | 12 (3, 48) | | 0.851 | 0 |

AUROC - area under the receiver operating characteristic, CI - confidence interval, RR - relative risk, ATB - antibiotic, AST - aspartate aminotransferase, ALT - alanine aminotransferase, LDH - lactate dehydrogenase. Statistical significant values are marked with Bold. We calculated *AUROC for quantitative variables and **RR for qualitative dichotomous variables
